# Draft Genome Sequence of *Streptomyces phaeoluteigriseus* DSM41896

**DOI:** 10.1128/genomeA.00371-17

**Published:** 2017-05-25

**Authors:** Jana K. Schniete, Talal S. Salih, Lis Algora-Gallardo, Tiago Santos, Sara Filgueira-Martinez, Paul R. Herron

**Affiliations:** a Strathclyde Institute of Pharmacy and Biomedical Sciences, University of Strathclyde, Glasgow, United Kingdom; b College of Sciences, University of Mosul, Mosul, Iraq

## Abstract

The draft genome for the type strain *Streptomyces phaeoluteigriseus* DSM41896 (ISP 5182) is reported. It was classified as a member of the *Streptomyces violaceusniger* clade; however, a polyphasic study showed it was a separate species based on its distinct spore morphology and 16S rRNA sequence. The genome sequence confirms it as a separate species.

## GENOME ANNOUNCEMENT

The genome of *Streptomyces phaeoluteigriseus* DSM41896 was sequenced. It was originally isolated from soil and incorrectly identified as a member of the *Streptomyces violaceusniger* clade and later described as a separate species based on phenotypic traits and differences in the 16S rRNA sequence (1).

The genomic DNA was extracted from a 3-day culture grown in ISP1 medium (2). The ISOLATE II Genomic DNA kit (Bioline, United Kingdom), including a prelysis lysozyme step, was used for isolation according to the manufacturer’s instructions. The library, targeting a 380-bp fragment size, was created using the Next Fast DNA Fragmentation & Library Prep Set for Ion Torrent (New England Biolabs, USA) and an E-Gel system (Invitrogen, USA) for the size selection. The quality assessment of the library was carried out using a dsDNA HS bioanalyzer kit (Agilent, USA).

The draft genome was sequenced using an Ion Torrent PGM at the Strathclyde Institute of Pharmacy and Biomedical Sciences (SIPBS), Glasgow, United Kingdom, using the Hi Q OT2 and Sequencing kit (Life Technologies, Inc., USA). Two runs were performed, one on a 318 chip and one on a 316 Chip v 2 type (Life Technologies, Inc., USA).

The reads were first assembled using SPAdes 3.9 (77 k-mer, single-cell mode, >1,000-bp length) as recommended for high-GC data sets (3). This was used to obtain an 81-gapped contig scaffold using MeDuSa (4), a multidraft-based analysis using 3 related species, *Streptomyces griseus*, *Streptomyces scabiei* and *Streptomyces hygroscopicus*, with full-genome sequences. The total number of contigs in the scaffold is 223, with the largest contig of 306,604 bp and an *N*_50_ value of 71,307. The total genome size is 8,629,293 bp with a G+C content of 71.5%. The assembly statistics were generated with QUAST 3.2 (5).

The annotation using the RAST server (6, 7) predicted 8,313 coding sequences (CDS), including 74 tRNA genes. The average nucleotide identity (ANI) calculator based on MUmer (8) was utilized and the closest relatives were *Streptomyces griseus* NRRL WC-3645 (86.87%), *Streptomyces stelliscabiei* NRRL B-24447 (86.28%), and *Streptomyces scabiei* 96-08 (86.26%) (9). These results indicate that this strain is a separate species and also support the findings from the polyphasic study of 2007 (1, 10).

The genome was analyzed for secondary metabolite biosynthetic gene clusters using the bioinformatic tool antiSMASH 3.0 (11), and a total of 38 putative secondary metabolite gene clusters were detected, 2 bacteriocins, 2 butyrolactones, 1 ectoine, 1 indole, 1 lantipeptide, 1 lassopeptide, 2 melanin clusters, 10 nonribosomal peptide synthetase (NRPS) clusters (2 type 1 polyketide synthases [t1pks]), 1 nucleoside, 3 siderophores, 5 terpenes, 2 type 1 polyketides, 3 type 2 polyketides, 1 type 3 polyketide, and 3 other clusters.

### Accession number(s).

This whole-genome shotgun project has been deposited at DDBJ/ENA/GenBank under the accession number MPOH00000000. The version described in this paper is version MPOH02000000.

## References

[1] Goodfellow M, Kumar Y, Labeda DP, Sembiring L 2007 The *Streptomyces violaceusniger* clade: a home for streptomycetes with rugose ornamented spores. Antonie Van Leeuwenhoek 92:173–199. doi:10.1007/s10482-007-9146-6. 17407000

[2] Shirling E, Gottlieb D 1966 Methods for characterization of *Streptomyces* species. Int J Syst Bacteriol 16:313–340.

[3] Bankevich A, Nurk S, Antipov D, Gurevich AA, Dvorkin M, Kulikov AS, Lesin VM, Nikolenko SI, Pham S, Prjibelski AD, Pyshkin AV, Sirotkin AV, Vyahhi N, Tesler G, Alekseyev MA, Pevzner PA 2012 SPAdes: a new genome assembly algorithm and its applications to single-cell sequencing. J Comput Biol 19:455–477. doi:10.1089/cmb.2012.0021. 22506599 PMC3342519

[4] Bosi E, Donati B, Galardini M, Brunetti S, Sagot MF, Lió P, Crescenzi P, Fani R, Fondi M 2015 Medusa: a multidraft based scaffolder. Bioinformatics 31:2443–2451. doi:10.1093/bioinformatics/btv171. 25810435

[5] Gurevich A, Saveliev V, Vyahhi N, Tesler G 2013 QUAST: quality assessment tool for genome assemblies. Bioinformatics 29:1072–1075. doi:10.1093/bioinformatics/btt086. 23422339 PMC3624806

[6] Aziz RK, Bartels D, Best AA, DeJongh M, Disz T, Edwards RA, Formsma K, Gerdes S, Glass EM, Kubal M, Meyer F, Olsen GJ, Olson R, Osterman AL, Overbeek RA, McNeil LK, Paarmann D, Paczian T, Parrello B, Pusch GD, Reich C, Stevens R, Vassieva O, Vonstein V, Wilke A, Zagnitko O 2008 The RAST Server: rapid annotations using subsystems technology. BMC Genomics 9:75. doi:10.1186/1471-2164-9-75. 18261238 PMC2265698

[7] Overbeek RA, Olson R, Pusch GD, Olsen GJ, Davis JJ, Disz T, Edwards RA, Gerdes S, Parrello B, Shukla M, Vonstein V, Wattam AR, Xia F, Stevens R 2014 The SEED and the rapid annotation of microbial genomes using subsystems technology (RAST). Nucleic Acids Res 42:D206–D214. doi:10.1093/nar/gkt1226. 24293654 PMC3965101

[8] Kurtz S, Phillippy A, Delcher AL, Smoot M, Shumway M, Antonescu C, Salzberg SL 2004 Versatile and open software for comparing large genomes. Genome Biol 5:R12. doi:10.1186/gb-2004-5-2-r12. 14759262 PMC395750

[9] Richter M, Rosselló-Móra R, Oliver Glöckner F, Peplies J 2016 JSpeciesWS: a web server for prokaryotic species circumscription based on pairwise genome comparison. Bioinformatics 32:929–931. doi:10.1093/bioinformatics/btv681. 26576653 PMC5939971

[10] Figueras MJ, Beaz-Hidalgo R, Hossain MJ, Liles MR 2014 Taxonomic affiliation of new genomes should be verified using average nucleotide identity and multilocus phylogenetic analysis. Genome Announc 2(6):e00927-14. doi:10.1128/genomeA.00927-14. PMC4256179 25477398

[11] Medema MH, Blin K, Cimermancic P, de Jager V, Zakrzewski P, Fischbach MA, Weber T, Takano E, Breitling R 2011 antiSMASH: rapid identification, annotation and analysis of secondary metabolite biosynthesis gene clusters in bacterial and fungal genome sequences. Nucleic Acids Res 39:W339–W346. doi:10.1093/nar/gkr466. 21672958 PMC3125804

